# Exploring colorectal cancer survivors’ perspectives on improving care delivery and the role of e-health technology: a qualitative study

**DOI:** 10.1007/s00520-023-08007-8

**Published:** 2023-08-31

**Authors:** Liza van Deursen, Jiska J. Aardoom, Eva E. Alblas, Jeroen N. Struijs, Niels H. Chavannes, Rosalie van der Vaart

**Affiliations:** 1 Department of Quality of Care and Health Economics, Center for Nutrition, Prevention, and Health Services, National Institute for Public Health and the Environment, Antonie Van Leeuwenhoeklaan 9, Bilthoven, 3721 MA the Netherlands; 2 Department of Public Health and Primary Care, Leiden University Medical Center, Leiden, the Netherlands; 3 National eHealth Living Lab, Leiden, the Netherlands; 4 Health Campus The Hague, Department of Public Health and Primary Care, Leiden University Medical Center, The Hague, the Netherlands

**Keywords:** E-health, Digital, Telemedicine, M-health, Colorectal cancer, Quality, Care pathway, Clinical pathway, Qualitative research, Patient journey

## Abstract

**Purpose:**

The purpose of this study was to gather insights from colorectal cancer (CRC) survivors on how to improve care for CRC survivors and how e-health technology could be utilized to improve CRC care delivery.

**Methods:**

Three semi-structured focus groups were held with sixteen CRC survivors. To initiate the discussion, an online registration form and two vignettes were used. The data was analyzed using the framework method.

**Results:**

Based on survivors’ experiences, five themes were identified as opportunities for improving CRC care delivery. These themes include better recognition of complaints and faster referrals, more information as part of the care delivery, more guidance and monitoring of health outcomes, more collaboration between practitioners, and more attention for partners and relatives. In addition, survivors expressed opportunities for using e-health to facilitate information provision, improve communication, and monitor survivors’ health conditions.

**Conclusion:**

Several suggestions for improvement of CRC care delivery were identified. These often translated into possibilities for e-health to support or improve CRC care delivery. The ideas of survivors align with the vast array of existing e-health resources that can be utilized to enhance CRC care delivery. Therefore, the next step involves addressing the implementation gap between the needs of stakeholders, such as CRC survivors and healthcare providers, and the e-health tools currently available in clinical practice.

**Supplementary information:**

The online version contains supplementary material available at 10.1007/s00520-023-08007-8.

## Introduction

Colorectal cancer (CRC) is the second most frequently diagnosed cancer in Europe, with 520,000 new cases reported in 2020 [1]. Although CRC continues to be the second deadliest cancer in Europe [1], the mortality rate is decreasing in Western countries due to better screening and more advanced treatment options, resulting in earlier detection and treatment [2, 3]. In the Netherlands, 66% of individuals diagnosed with CRC survive for 5 years or more post-diagnoses, compared to the European average of 57% [4, 5]. This could be partially attributed to the country’s effective national screening program, which received a response rate of 76% between 2014 and 2018 [6].

The field of CRC care, like other areas of healthcare, faces several significant challenges. These challenges include a shortage of personnel, an increase in cancer incidence due to population growth and an aging population, and increasingly complex care due to the expansion of treatment options [7, 8]. These challenges have resulted in an increasing demand for CRC care with a broader scope. Additionally, as survival rates improve, ongoing care after medical treatment, such as through regular check-ups and supportive treatments, is essential in helping cancer survivors cope with the challenges they face in their daily lives [9]. Consequently, reforming the way care is delivered has become necessary.

The use of information and communication technology (ICT) in the healthcare field [10], known as e-health, has shown great potential in improving care delivery for CRC survivors. For example, digital communication and online information services are already being used throughout the CRC care pathway. Incorporating e-health into CRC care can significantly enhance care delivery and support. For instance, post-operative telemonitoring can help track a cancer patient’s health status, detect relapse early, and reduce healthcare costs while preventing complications [11–13]. Patient portals and digital applications can facilitate communication between survivors and healthcare providers and allow access to relevant health information and resources to improve survivors’ self-management. The transformative potential of e-health has been previously described by Wallace [14], highlighting its ability to answer critical cancer-related queries at a faster pace, greater scale, and broader scope.

However, the potential of e-health interventions in healthcare is yet to be fully realized due to limited adoption [15–17]. According to the Diffusion of Innovations Theory of Rogers, e-health services must meet end-users’ needs for successful implementation [18]. Therefore, it is recommended to identify healthcare providers’ and CRC survivors’ needs and preferences before developing and implementing e-health services for CRC care [18–20]. Previous research has examined the views of Dutch healthcare providers and managers on how e-health could improve CRC care [21]. This study identified several opportunities for improvement, such as using e-health applications to support survivors in the pre-habilitation program and implementing digital consultation hours to increase healthcare access and reduce unnecessary hospital visits.

Presently, there is limited understanding of the opinions of CRC survivors regarding the use of e-health applications. Most studies have only examined specific interventions [22]. CRC survivors are individuals who have been diagnosed with CRC and continue to live with the disease [23]. They have unique experiences and perspectives and face many challenges, such as fatigue, sleep difficulty, fear of recurrence, anxiety, depression, negative body image, and sensory neuropathy. They may also experience gastrointestinal problems, urinary incontinence, and sexual dysfunction [24].

This study aimed to identify areas where CRC care delivery could be improved according to the experiences of CRC survivors. Moreover, the study aimed to gather insights from CRC survivors on how e-health technology could improve CRC care delivery.

## Methods

### Study design

For this study, a phenomenological approach was used [25]. This research method involves examining a phenomenon from the perspective of those who have experienced it, to define its essence and significance [26]. To examine the experiences of Dutch CRC survivors with CRC care delivery, semi-structured online focus groups were conducted. Focus groups encourage participant interaction, promoting the emergence of diverse perspectives and social interactions [27], which leads to a deeper understanding of their experiences. A semi-structured format was used to allow for more flexible questioning and exploration of topics and to delve deeper into the responses provided by participants [28, 29]. The focus group protocol was crafted by the primary author (LvD), predominantly drawing from previous relevant research [21, 30]. Collaborative contributions from RvdV, JJA, and JS enriched the protocol through insightful feedback and suggestions shared during interdisciplinary team meetings. This iterative refinement process aimed to strengthen the depth and scope of the topic guide. An overview of the focus group protocol can be found in Supplementary file 1.

### Data collection

To be enrolled in the study, participants had to meet all the following eligibility criteria:

- Be a CRC survivors aged 18 years or older diagnosed with cancer stages I–IV.
- Currently receiving treatment or have undergone treatment within the past 5 years.
- Possess a good command of the Dutch language.
- Have utilized at least one form of e-health throughout the CRC care pathway.

Convenient sampling was used to gather participants, with online invitations disseminated through various channels such as patient associations, research institutions like the Netherlands Comprehensive Cancer Organization, and the LinkedIn pages of the researchers and their organizations. Furthermore, the invitation was also posted in Dutch Facebook groups for cancer survivors.

The call to participate included a link to a registration form to collect the following information: demographic characteristics, cancer stage at diagnosis, treatment period, type of treatment, and hospital, and information on the types of e-health used throughout the CRC care pathway. Participants were also asked to provide two examples of instances where they saw room for improvement in CRC care delivery based on their personal experiences, which served as input for the focus group discussion.

After completing the form and when eligible for participation, applicants received an email with an information letter and a date planner to schedule the focus groups. To participate, individuals had to confirm via email that they had read the information letter and provided written consent.

Initially, 28 individuals signed up, all meeting the inclusion criteria, but nine dropped out before an interview could be scheduled. Therefore, three focus groups were planned with nineteen participants. Three individuals did not show up during the focus group meeting, resulting in sixteen participants.

The online focus groups were conducted in Dutch by two female researchers with a background in psychology and trained in focus group discussions (LvD, MSc and RvdV, PhD) until data saturation was reached (i.e., when new incoming data produced little or no further information to address the research question). The focus groups were video-recorded for analysis purposes.

### Content of the focus groups

During the focus group, two main topics were discussed. The first one was about opportunities to enhance CRC care delivery throughout the CRC care pathway. The second topic was about using e-health to improve CRC care delivery. The term “CRC care pathway” refers to how Dutch medical specialist care for CRC survivors is typically organized. This pathway includes referral, diagnosis, treatment, aftercare, and sometimes, palliative care [17]. A detailed explanation of each phase in a standard CRC care pathway can be found in Supplementary file 2.

The first topic was introduced by summarizing participants’ answers to the open-ended question in the registration form. Also, the care pathway was shown to help participants consider their experiences throughout all stages of the care pathway. For the second topic, an overview slide was shown with a definition of e-health and an explanation of different e-health categories. These categories were based on a framework of Nictiz, a Dutch knowledge center for national ICT applications in healthcare [31], adapted to categories of technology and digital health services relevant to CRC survivors. The e-health categories used included digital communication, telemonitoring, online information services, personal health environment, self-monitoring, and patient portals. More information about these categories and their explanations can be found in Supplementary file 3.

To start the conversation on the first topic, two hypothetical short stories were presented as examples of how e-health could improve CRC care delivery. These stories, known as vignettes [32], provided practical examples of how e-health can enhance different phases of the care pathway. The vignettes were based on previous research on how healthcare professionals perceive the potential benefit of e-health throughout the CRC care pathway [21]. The stories described the use of different e-health technologies in different phases of the care pathway. The first vignette narrated a woman’s experience choosing between a digital intake, comprising online information and a digital questionnaire, or a regular face-to-face intake before undergoing an endoscopy. The second vignette described how a man’s recovery from surgery was monitored by his doctor through a smart patch and daily digital questionnaires. The complete version of the vignettes can be found in Supplementary File 4. Following the discussion of the vignettes, the researchers posed specific questions to encourage a more expansive conversation beyond the content of the vignettes to gain a broad insight into the participants’ experiences during their care process.

### Data analysis

Data analyses were conducted in MAXQDA 2022 software [33]. The framework method was used for data analysis [34], a qualitative content analysis approach highly adaptable for studies that aim to generate themes. This method is suitable for semi-structured focus groups, and offers clear steps to follow, and produces highly structured outputs of summarized data, making it beneficial when multiple researchers are involved in a project [34]. Each step of the framework method was followed throughout the analysis, which is detailed below.

Firstly, the focus groups were transcribed word-for-word, and the researchers familiarized themselves with the data. Next, two researchers, LvD and EA, independently coded the initial focus group data using deductive coding based on the protocol’s topics and inductive coding based on emerging topics from the data. These codes were compared until the researchers agreed on a working analytical framework (i.e., developing a thematic framework), which was used to analyze the remaining data (i.e., indexing). Any discrepancies were discussed until a consensus was reached. Finally, the researchers created a framework matrix to summarize the data from each focus group per category (i.e., charting) and interpreted the data (i.e., mapping and interpretation). Data saturation was achieved regarding the identified themes. The relevant quotes were translated into English and presented in the following format: survivor number, gender, age in years, and cancer stage number.

### Rigor and quality assurance

To ensure the accuracy and quality of the research findings, we utilized the Consolidated Criteria for Reporting Qualitative Research (COREQ) checklist [35]. A completed version of the checklist can be found in Supplementary file 5. Furthermore, the coding scheme can be found in Supplementary file 6.

## Results

### Participants’ demographics

Table 1 provides an outline of the demographic details of the focus group members, totaling sixteen participants. There were two focus groups with five participants, and one with six participants. The average age of the participants was 55 years (SD = 9, range 39–70).

**Table 1:** Characteristics of participants (*N* = 16)

Characteristic	*n*	(%)
Sex		
Male	8	(50)
Female	8	(50)
Educational level		
Secondary education	1	(6)
Post-secondary vocational education (MBO)	2	(12)
Higher Professional Education (HBO)	7	(44)
Academic education (WO)	6	(38)
Treatment period		
Currently undergoing treatment	2	(12)
Finished treatment < 1 year ago	6	(38)
Finished treatment 1–3 years ago	5	(31)
Finished treatment 4–5 years ago	3	(19)
Type of treatment^1^		
Operation	16	(100)
Chemotherapy	10	(62)
Radiotherapy	4	(25)
Experimental therapy	1	(6)
Targeted therapy	1	(6)
Palliative care	1	(6)
Type of hospital		
Academic	5	(31)
Non-academic	11	(69)
Stage of cancer at diagnosis		
Stage 1	3	(19)
Stage 2	4	(25)
Stage 3	7	(44)
Stage 4	2	(12)

^1^Some of the participants received multiple forms of treatment

### Participants’ views on opportunities to improve CRC care delivery

This section outlines five opportunities to enhance CRC care delivery based on participants’ personal experiences. At least two participants have mentioned these opportunities.

- **1. Need for better recognition of complaints and faster referrals**

Some participants had negative experiences with the recognition of complaints and the general practitioner’s speed of referral to the hospital for initial diagnosis of CRC. Among multiple participants, the complaints were initially not recognized as CRC symptoms. In some cases, this was likely due to their age, as two participants explained that they were much younger than average CRC survivors.

“Due to my age, it wasn’t noticed very quickly. I was told that I was too young, that it’s an old man’s trouble.” (Survivor 1, female, 52, stage 2).

Another participant mentioned that her general practitioner believed that hemorrhoids were the cause of her complaints.

- **2. Need for more information as part of the care delivery**

Several participants reported that the medical-related information provided throughout different stages of the care pathway was not satisfactory or incomplete. One participant even received contradictory information during diagnosis. Others expressed a desire for more information regarding treatment options, side effects, recovery, medication, nutrition, physical fitness, coping mechanisms, mental health, reintegration, and palliative care.

“At the time of diagnosis, we received very contradictory information, about which we later complained. Clear and transparent communication is very important.” (Survivor 2, male, 43, stage 2).

Some respondents reported that the amount of personalized and in-depth information they received was directly related to how assertive they were in seeking it out. Certain participants only received customized information when they specifically requested it. Moreover, some participants felt reluctant to contact healthcare professionals with any questions they had during and after treatment.

Furthermore, participants reported needing clarification about which health professional to approach for specific concerns. They also missed having a designated point of contact for their questions.

- **3. Need for more guidance and monitoring of health outcomes**

During their chemotherapy treatment, many participants felt unsure of how to monitor their health and experienced unfamiliar physical issues and changing side effects. They expressed a desire for more comprehensive guidance and support on these matters. Additionally, some participants felt that they were not being closely monitored after being discharged from the hospital after treatment to recover at home and wished for better monitoring of their physical and mental health during this phase. For instance, one participant mentioned that the hospital frequently called her during treatment to check in, but the calls stopped after the treatment. She would have appreciated continued monitoring during aftercare.

“The aftercare following the surgical removal procedure is subpar. There seems to be a lack of guidance and support, without any inquiry into my needs. For instance, I have not received any psychological counseling or follow-up monitoring after being discharged from the hospital” (Survivor 2, male, 43, stage 2).

- **4. Need for more intra- and interdisciplinary collaboration**

According to some participants, practitioners could have collaborated more effectively within and across disciplines. They reported that practitioners did not always consult with each other to solve unexpected problems and indicated a lack of alignment between hospitals or other healthcare organizations. Furthermore, they said that various practitioners within the hospital did not seem aware of each other’s activities. This resulted in an inadequate transfer of information and survivors feeling overlooked. A suboptimal digital infrastructure partly caused this. For example, a participant mentioned she was treated in multiple hospitals, but practitioners could not access each other’s records.

“There are an awful lot of healthcare providers, and sometimes I feel like they all operate in their own little box. I know there is always interdisciplinary collaboration, but I do sometimes get the impression that things get a bit disjointed and I’m not sure who to turn to.” (Survivor 1, female, 52, stage 2).

- **5. Need for attention for partners and relatives**

Finally, some participants said they wished their family and relatives had received more attention. They believed their significant others should have had the opportunity to share their experiences and receive help and support from peers or professionals on coping with problems they encountered.

“There should be more attention for the loved ones; being sick is not something you do alone, but it also has much impact on your family.” (Survivor 3, male, 63, stage 3).

### Participants’ use and perception of e-health

Throughout the CRC care pathway, participants used various e-health categories. The most commonly used were online information services (88%) and patient portals (62%). Telemonitoring, on the other hand, was only used by one (6%) participant. Table 2 contains additional information on the use of different technologies by the participants.

**Table 2:** E-health categories used by participants throughout the CRC care pathway (*N* = 16)

E-health category	*n*	(%)
Online information services	14	(88)
Patient portal	10	(62)
Digital communication	7	(44)
Self-monitoring	3	(19)
Telemonitoring	1	(6)

### Participants’ views on how e-health could be used to improve CRC care delivery

In this section, participants’ views on how e-health can enhance CRC care delivery are described. Most ideas directly build on participants’ general suggestions to improve CRC care delivery, as described above.

- **1. The use of digital tools could help provide more comprehensive information**

Participants indicated they would have liked to receive more information throughout the care pathway. They responded positively to replacing a face-to-face intake for an endoscopy with a digital intake, which includes online questionnaires and digital information about the endoscopy in the patient portal (presented in Vignette 1). This would allow them to access information at their convenience and review it again if needed. Additionally, the information can be provided in various forms, such as animations or written communication. It would also save time and eliminate the need to travel to the hospital.

“Naturally, I received flyers about what a colonoscopy looks like, what a certain operation looks like, and then you read that. However, I prefer visual information. For me, an animation or something would be more informative. I would have appreciated seeing a link or video on my portal about appropriate treatments and operations.” (Survivor 4, female, 47, stage 3).

However, participants expressed the need for flexibility in the intake process, as not all survivors are able or willing to use digital options. Technical support should also be easily accessible to survivors, and they should be able to provide any specific information they deem relevant to healthcare workers.

Participants report that digital information could be used to better support their inquiries during treatment and aftercare. Specifically, they would appreciate having access to information about treatment options, potential side effects, residual clinical damage, the recovery process, nutritional guidance, physical fitness, coping with the disease, mental health(care), and palliative care. This information could be offered through a reliable source such as the patient portal. Some participants were aware that relevant information was already available in the patient portal of their hospital, but not all participants were informed about this.

“Make sure there is a platform where people can find the information. What are your options? What kind of support is provided, such as oncological physiotherapy? Diet? Dealing with pain? You name it.” (Survivor 5, female, 65, stage 3).

- **2. Increasing digital communication with healthcare providers could help provide more comprehensive information**

Participants expressed that they would have found it helpful to be able to communicate with their healthcare providers directly through digital channels. They suggested that this option should be made more easily accessible to lower the threshold for asking questions, as many participants felt hesitant to disturb their providers due to their high workload. To address this concern, participants suggested introducing a digital consultation hour, where survivors can save their inquiries until a specified time to communicate with their providers. Another suggestion was to have case managers refer survivors to the appropriate practitioner for digital communication, which would help clarify the responsibilities of each healthcare provider.

“Usually, when you have a question or something bothering you, you try to approach someone. But if I knew that every day between two and three, you could ask questions digitally or have a chat function and then maybe even say, ‘Let’s have a phone call,’ that would be really nice. Especially for questions that can wait a few hours.” (Survivor 1, female, 52, stage 2).

- **3. Telemonitoring could provide more guidance and monitoring**

Participants expressed their desire to have guidance and be monitored during their recovery at home post-hospitalization. Telemonitoring after surgery, as presented in Vignette 2, was seen as a beneficial option to fulfill this need. Participants believed telemonitoring would provide a reassuring check-in during the first weeks of recovery at home after surgery. This would offer a sense of security, particularly since multiple participants were unsure about their ability to self-monitor during this phase.

“But you don’t know what hit you. You are terribly ill, and you’re so upset that you can’t monitor yourself. If you, for example, develop a fever, you must contact the hospital immediately. That is one of the things you have to monitor yourself, but are you aware of that? I’m not.” (Survivor 1, female, 52, stage 2).

Participants also expressed a desire to remain in the hospital for a certain period after surgery to receive care and advice and ask questions. Although they were open to the idea of leaving the hospital early and recovering at home, some participants were concerned that an early release combined with telemonitoring would be a cost-cutting measure instead of a survivor-focused improvement.

One participant suggested sharing telemonitoring data between the physiotherapist and the hospital for better recovery support. Furthermore, participants thought it would be valuable to access the telemonitoring data themselves. They believed that having insight into their health data could provide reassurance or confirm suspicions of abnormalities, making it easier to contact the doctor. Furthermore, participants presumed that it would facilitate self-management and motivate survivors to work on their recovery by viewing data, such as physical activity data. However, it was noted that adequate digital skills are needed for survivors to use and benefit from telemonitoring tools.

Another suggestion for monitoring after treatment was to use an app that allows survivors to enter specific symptoms and subsequently receive automatically generated feedback on whether these are usual or unusual side effects.

- **4. Enhanced sharing of electronic data could facilitate greater collaboration among professionals**

Participants expressed a desire for their practitioners to collaborate more within and across disciplines but noted that the current digital infrastructure posed challenges. They suggested that healthcare practitioners should be able to read summaries of consultations and reports of examinations and treatments. In other words, to facilitate practitioners’ collaboration, they should be able to access each other’s records of survivors with whom they have a treatment relationship.

“I was treated in two separate hospitals. So, I have a digital record in both hospitals. The doctors cannot access each other’s records, so I have additional scans in hospital A, for example, since they were first made in hospital B. And there is no communication between those two hospitals. And I find that very difficult because then you are with the nurse, and you say, yes, but that is in that record. Yeah, she can’t access it. So, then I have to open the record of hospital A myself to show it to the nurse in hospital B.” (Survivor 5, female, 65, stage 3).

- **5. Digital platforms could support peer-to-peer contact**

Several participants shared their experiences with peer-to-peer communication, both online and offline. Some had attended physical meetings organized by patient organizations and found them beneficial. Others found it helpful to actively participated in closed support groups on social media initiated by, for example, patient organizations. Some participants suggested that healthcare providers should refer patients to these digital groups. Another group of participants found blogs and responses valuable sources of practical advice and a way to feel recognized and acknowledged. However, some participants expressed concerns about privacy and safety on social media and blogs and suggested that healthcare providers should facilitate peer-to-peer communication within the healthcare sector.

“I think that contact with peers should also be facilitated. That is very important. I would never choose a social media platform construction because of safety, privacy, you name it. Even when that is a closed support group, all your information will still go public.” (Survivor 3, male, 63, stage 3).

Several participants suggested that facilitating peer-to-peer contact could provide additional support to family members and relatives in response to the expressed need for more attention to be given to them. They proposed a forum where partners and relatives could exchange experiences, ask questions, discuss problems, and provide advice to one another.

## Discussion

The study’s primary goal was to gather insights from CRC survivors’ regarding areas where the delivery of CRC care could be improved. The study identified five areas for improvement: (1) better recognition of complaints and faster referrals, (2) more information during multiple phases of the care pathway, (3) more guidance and monitoring during aftercare, (4) more collaboration between practitioners, and (5) more attention for partners and relatives.

Identified improvement opportunities covered all phases of the care pathway. Participants frequently mentioned they needed more information and guidance throughout the care pathway, especially after treatment. Survivors require information and guidance on topics that are not directly related to their treatment, such as nutrition, disease coping, and reintegration into working life. Beuken et al. (2022) also noted that cancer care in Dutch hospitals currently focuses on medical treatment by medical specialists. They do not always refer to additional (after)care interventions that match survivors’ wishes and needs [36]. Another finding, that survivors do not always see optimal cooperation between healthcare providers, is in line with a vision document from the Dutch Taskforce Cancer Survivorship Care [37]. They emphasize that continuity and coordinated cohesion in cancer care are essential starting points and that more cooperation within and between the chain is needed. The document also highlights the role of data exchange in achieving this goal [37].

The study’s second objective was to gather insights from CRC survivors on how e-health technology could be utilized to enhance CRC care delivery. Participants identified various ways e-health could support survivors’ needs and improve CRC care delivery. Most ideas for using e-health build upon the abovementioned themes for improving CRC care delivery. Most ideas aimed to either facilitate information provision (e.g., online information in the patient portal to access information when needed), improve communication (e.g., facilitating adequate electronic data sharing among practitioners and online platforms for peer-to-peer contact), or monitor survivors’ health conditions (e.g., using telemonitoring tools for practitioners to better monitor survivors’ recovery after surgery and provide them with a sense of safety).

The study’s findings on how e-health can improve CRC care delivery align with the three domains of the e-health framework developed by Shaw and colleagues [38]. These domains include using e-health technologies to monitor, track, and inform about health; communicating between stakeholders in health; and collecting, managing, and using health data sources. Shaw and colleagues also argue that a distinctive feature of e-health is its fluid boundaries; therefore, the domains can overlap. The findings of our research show this as well. For example, a telemonitoring tool can serve both as a health data collection tool and a means of informing survivors about their monitoring data. Similarly, digital peer-to-peer contact tools can facilitate peer communication and inform survivors about their health [39, 40].

The findings of the current study on e-health improvement opportunities overlap with those of a comparative study conducted among healthcare professionals [21]. For example, survivors and professionals favored a digital intake to prepare for endoscopies and better use of online information and digital questionnaires. Additionally, both groups reported that (health) data exchange between healthcare professionals should be improved. Furthermore, both survivors and professionals made several critical remarks on using e-health, such as not all e-health technology being suitable for every survivor. However, survivors also proposed ideas for using e-health that did not emerge during conversations with professionals, such as using digital platforms for peer-to-peer contact with partners and relatives and increased use of digital information in the patient portal to support inquiries during aftercare. Survivors also mentioned specific necessities, such as being able to ask questions or add comments to the digital questionnaires in a digital intake for an endoscopy. This shows that survivors have unique perspectives, relevant ideas, and preconditions based on their experiences, which should be considered when considering e-health to improve the healthcare system.

Many evidence-based tools are already available that can accommodate stakeholders’ needs, as mentioned in this study, for example, online interventions to support, guide, and monitor survivors during aftercare [30, 41–43] and a digital intake for an endoscopy to receive complete information [44]. However, they do not seem to be used frequently or are not scaled up adequately. In other words, supply and demand often do not find each other. Thus, an important question is how these tools can be more effectively implemented on an organizational level. Relevant parties, such as healthcare organizations, insurers, and policymakers, should focus on closing the gap between the needs of stakeholders (i.e., CRC survivors and healthcare providers) and the tools already available. Other relevant questions are whether survivors and healthcare providers are already aware of available tools, whether they are willing and capable of using them, and how these factors could be improved.

Future research should also focus on what is needed to adequately implement the ideas for e-health use mentioned by survivors and healthcare professionals and the requirements that apply. This can be accomplished, for example, through practice-oriented action research that considers the specific context. Additionally, it would be useful to explore to what extent this study’s results fit different cancer care types to determine to what extent current results are generalizable to other kinds of (cancer) care and what else is needed.

It should be noted that this study had some limitations. Firstly, most participants were relatively young, highly educated, and likely more digitally proficient than the average population of CRC survivors. However, participants did provide suggestions for less digitally skilled survivors. Secondly, the *r* sample size was relatively small. However, data saturation was achieved on the discussed topics after three focus group discussions. Finally, only two types of clinical situations and e-health technology were discussed in the vignettes, which may have limited the discussion’s focus. However, the vignettes were valuable since they did not require the participants to have in-depth theoretical knowledge of the study’s subject [45], and they inspired participants to start the conversations. Furthermore, the researchers actively encouraged a broader discussion beyond the content of the vignettes.

## Conclusion

CRC survivors possess a unique outlook on enhancing the delivery of CRC care and how e-health can aid in this. Drawing from their personal experiences, they offer valuable suggestions for improving CRC care delivery and effectively using e-health applications. They also highlight important considerations and limitations regarding e-health research and implementation in daily practice. Their ideas regarding the use of e-health are diverse and intended to facilitate information provision, communication enhancement, and remote monitoring of survivors. Since numerous e-health tools already exist to cater to the needs of survivors, it is crucial to explore ways to match supply and demand better.

## Supplementary information

Below is the link to the electronic supplementary material.

Supplementary file 1. Focus group protocol (DOCX 35 KB)

Supplementary file 2. CRC care pathway phases (DOCX 28 KB)

Supplementary file 3. Description of e-health categories (DOCX 25 KB)

Supplementary file 4. Vignettes (DOCX 493 KB)

Supplementary file 5. COREQ checklist (DOCX 28 KB)

Supplementary file 6. Coding scheme (XLSX 18 KB)

## Data Availability

The data that support the findings of this study are available from the corresponding author on reasonable request.
