# Supplementary material for: Exploring colorectal cancer survivors’ perspectives on improving care delivery and the role of e-health technology: a qualitative study
Source: Support Care Cancer. 2023 Aug 31;31(9):544. doi: 10.1007/s00520-023-08007-8 (PMC10471668; doi:10.1007/s00520-023-08007-8)
Supplement: Supplementary file 1 — Focus group protocol (DOCX 35 KB) [file 520_2023_8007_MOESM1_ESM.docx]

**Focus group protocol [translated from Dutch]**

| Introduction, Purpose, and Ground Rules 5 minutes |
| --- |

*Share slide with logos of organizations and discussion purpose*

**Welcome and introductions**

First of all, thank you very much for joining us today. We greatly appreciate your time and participation in this conversation. I would like to start by introducing myself and explaining the background and purpose of the research. After that, I would like to hear more about each of you.

My name is Liza van Deursen, and I work as a PhD candidate for the E-healthmonitor. The E-healthmonitor is a joint project of the National Institute for Public Health and the Environment, Nivel and the National E-health Living Lab (part of Leiden University Medical Center). The project started in 2021, commissioned by the Dutch . The aim of the project is to map the use of digital healthcare and learn how digital healthcare can contribute to patient care.

I am here with my colleague Roos van der Vaart, who is the project leader for the E-healthmonitor. During this conversation, she will be present to take notes in the background and ask additional questions if needed.

*Share slide with research objectives and themes*

**Research objective**

In previous research, we spoke with healthcare providers involved in the care of people with colorectal cancer. We discussed with them the possibilities for improvement in colorectal cancer care and the role of digital healthcare in this context.

In this group conversation, we want to talk with you about the improvement opportunities you see in colorectal cancer care and your perspectives on digital healthcare.

Our goal is to hear from people with colorectal cancer about what can be improved and how digital healthcare can support that.

The following topics will be discussed in this conversation:

1. The most important improvement opportunities you see in colorectal cancer care based on your own experiences; and
2. The potential of digital healthcare to contribute to improving colorectal cancer care.

So, we are not focusing on shortcomings but rather on how we can make the care for people with colorectal cancer as good as possible.

*Share slide with explanation of group conversation*

**Ground rules**

I would like to explain how we will conduct the conversation:

• In this group conversation, I invite you all to share any ideas, opinions, and personal experiences you have. What do you find important? What are your ideas, concerns, and suggestions? There are no right or wrong answers. It's about what you think and why you think that way.

• I encourage you to engage in discussion with each other as much as possible. You can also ask each other questions. You are in charge of this conversation.

• The role of the facilitator is to ask questions, probe further when necessary, and occasionally guide or redirect the discussion if needed.

• I kindly ask you to raise your hand if you want to speak. I, as the facilitator, will give people the floor. I will try to give everyone an opportunity to speak.

• The conversation will last approximately 1.5 hours.

• I suggest using "you" and "your" to make it less formal.

• An anonymous report will be made of this meeting, and as previously announced, a video recording will be made for this report. The recording will be deleted afterward.

• Based on the group conversations, we will write an article. If you are interested, you can receive a copy of the article. We will note this down after the conversation.

Finally, I want to emphasize that we realize that each of you has your own story. You have all been through a lot, and I can imagine that there is much to say about it. Unfortunately, due to time constraints, we may not always be able to delve into it as much as we would like. This is because we have specific questions that we need to ask. I respectfully ask you to understand this if we sometimes have to interrupt your story earlier than desired.

• Are there any questions before we begin?

• Then I will start the video recording now.

| Introduction Round 10 minutes |
| --- |

*Share slide with introductory questions*

• Can you share your name, age, and when you received the diagnosis

| Core Questions 60 minutes |
| --- |

*Share slide with theme 1*

Now I would like to start with the first theme of this conversation, which is improvement opportunities in colorectal cancer care. We will spend approximately 15 minutes on this part.

*Share slide with colorectal cancer care pathway*

**Topic 1: Colorectal Cancer Care Pathway Improvement Opportunities (15 minutes)**

First, I'm curious about the improvement opportunities you see in colorectal cancer care based on your experiences. We will think about this in relation to the care pathway that most people with colorectal cancer go through. What does this pathway look like? To inspire you, you will see a diagram of the different phases in the care pathway on the slide.

We asked you beforehand to write down 2 or 3 experiences of the care you received where you thought, "This could be better." This could be about any phase in the care pathway. We will provide a brief summary of your ideas. Of course, this summary is not exhaustive. After this summary, we are interested in your reflections.

**Discussion on improvement opportunities based on experiences**

Could you give a brief response to this summary?

**Topic 2: The Role of Digital Healthcare in Improving Colorectal Cancer Care (35 minutes)**

*Share slide with theme 2*

Thank you for sharing your experiences. We now have a good initial understanding of the improvements you see in the colorectal cancer care pathway. Now I would like to move on to the second theme of this conversation: the role of digital healthcare in improving colorectal cancer care. First, a question for all of you:

*Share slide with question*

• What comes to mind when you hear the term 'digital healthcare'?

It's interesting to see your ideas about digital healthcare. We define digital healthcare as all information and communication technologies that support or improve healthcare.

*Share slide with examples of digital healthcare*

To give you an overview of what can be considered as digital healthcare, this slide shows some examples of categories of digital healthcare.

Now I would like to discuss with you how digital healthcare could have helped improve your care, based on your experiences. In other words, looking back on the care you received, how could digital healthcare have provided you with a better experience?

It's important to mention that we don't see digital healthcare as an end in itself, but rather as a means to improve care—for example, improving quality, providing more continuity, or saving time. We are curious about the possibilities you see for this.

To help you think about this, I would like to share two short stories with you as inspiration. These stories illustrate different digital healthcare technologies in various stages of the care pathway.

*Share slide with questions for the vignettes*

For each story, I would like you to consider two things:

1. What do you think about the way care is provided in this story?
2. Does this story or any part of it give you ideas about how the issues you encountered in your care process could be resolved or improved?

*Share slide with image of vignette 1 (and then the same for vignette 2)*

*Read vignette 1*

*Share slide with technologies in care pathway phase for vignette 1*

This slide briefly shows which technologies were mentioned in this story and in which phase. Now I would like to return to the questions I presented to you earlier.

1. What do you think about the way care is provided in this story?
2. Does this story or any part of it give you ideas about how the issues you encountered in your care process could be resolved or improved?

Probing question:

-Could these applications be valuable in another phase?

After the vignettes:

*Share slide with examples of digital healthcare*

We have discussed your ideas based on the vignettes. Finally, I would like to show you the slide with examples of digital healthcare again.

Let's reflect on your own experiences. Do you have any other ideas on how one of these technologies can improve healthcare?

Probing question:

- Looking at the slide with your own experiences, there were also mentioned improvement opportunities that (choose the ones that haven't been discussed much):

*Fall within the aftercare phase /

*Relate to communication and the doctor-patient relationship /

*Are about 'dealing with the disease' and 'rehabilitation';

Could digital healthcare play a role in those areas?

| Conclusion 5 minutes |
| --- |

We have now obtained a comprehensive overview of improvement opportunities in colorectal cancer care, and the potential for digital healthcare.

- Are there any points that we haven't discussed that you would like to mention?
- What did you think of the conversation?

| Follow-up and gratitude 5 minutes |
| --- |

We want to thank you once again for your cooperation.

We will process and analyze the conversations anonymously. The results will be published in a scientific article. Are any of you interested in receiving this article?

We will send you the gift card via email soon.

If you have any questions or additional contributions afterward, please feel free to contact us. My email address is provided on the slide.

**Gesprekshandleiding focus groepen mensen die leven met en na darmkanker [original text in Dutch]**

**Inleiding, doel en spelregels** **5 minuten**

**Delen slide met logo’s organisaties en doel gesprek**

*Welkom en voorstellen*
Allereerst hartelijk bedankt voor jullie komst vandaag. We waarderen het enorm dat jullie tijd hebben vrij gemaakt voor dit gesprek. Ik zou willen starten door mijzelf voor te stellen en de aanleiding en het doel van het onderzoek toe te lichten. Daarna kom ik graag terug bij jullie om te horen wie jullie zijn.

Mijn naam is Liza van Deursen en ik ben werkzaam als promovendus voor de E-healthmonitor. De E-healthmonitor is een gezamenlijk project van het RIVM, het National E-health Living Lab (NeLL), onderdeel van het LUMC, en Nivel. In 2021 is het project gestart, in opdracht van het ministerie van Volksgezondheid. Het project heeft als doel om de inzet van digitale zorg in kaart te brengen en te leren over hoe digitale zorg iets kan bijdragen aan de zorg.

Mijn persoonlijke drive om dit werk te doen is om te leren over hoe digitale zorg nou op zo’n manier kan worden ingezet dat het de zorg voor mensen met kanker écht verbeterd.

Ik ben hier samen met mijn collega Roos van der Vaart, zij is projectleider van de E-healthmonitor. Tijdens dit gesprek is zij aanwezig om op de achtergrond aantekeningen te maken en eventueel aanvullende vragen te stellen.

**Delen slide met doel OZ en thema’s**

*Doel onderzoek*
In eerder onderzoek hebben we gesproken met zorgverleners betrokken bij de zorg voor mensen met darmkanker. We spraken met hen over de verbetermogelijkheden in de darmkankerzorg en de rol van digitale zorg hierin.

In dit groepsgesprek praten we met jullie over de verbetermogelijkheden die jullie zien in de darmkankerzorg en jullie kijk op digitale zorg.

Ons doel is om van de mensen met darmkanker te horen wat er beter kan en hoe digitale zorg daarin kan ondersteunen.

De volgende onderwerpen zullen in dit gesprek aan bod komen:
1) de belangrijkste verbetermogelijkheden die jullie zien in de darmkankerzorg op basis van jullie eigen ervaringen;
2) de mogelijkheden van digitale zorg om bij te dragen aan het verbeteren van het darmkankerzorg.

We willen dus niet zo zeer kijken naar tekortkomingen maar naar: hoe kunnen we de zorg zo goed mogelijk maken voor mensen met darmkanker.

**Delen slide met toelichting groepsgesprek**

*Spelregels*Graag licht ik ten slotte nog wat toe over hoe we het gesprek aanpakken;

- In dit groepsgesprek wil ik jullie van harte uitnodigen om alle ideeën, meningen en persoonlijke ervaringen die je hebt te delen. Wat vind je belangrijk, wat zijn je ideeën, bedenkingen en vooral suggesties? Er zijn geen goede of foute antwoorden. Het gaat erom wat je vindt en waarom je dat vindt.
- Ik wil jullie aanmoedigen om zoveel mogelijk onderling te discussiëren. Je mag elkaar ook vragen stellen. Jullie zijn aan zet in dit gesprek.
- De rol van de gespreksleider is om vragen te stellen, door te vragen waar nodig en af en toe de discussie af te bakenen of bij te sturen indien nodig.
- Ik wil jullie vragen om je hand op te steken als je wat wilt zeggen. Ik zal als gespreksleider aan mensen het woord geven. k zal proberen om iedereen aan het woord te laten komen.
- Het gesprek duurt ongeveer 1,5 uur.
- Ik stel voor om ‘je’ en ‘jij’ te zeggen om het wat minder officieel te maken.
- Er wordt anoniem verslag gemaakt van de bijeenkomst en voor dit verslag wordt zoals vooraf aangekondigd een video-opname gemaakt. Deze wordt na afloop verwijderd.
- Op basis van de groepsgesprekken maken we een artikel. Als je belangstelling hebt kun je het artikel ontvangen. Dit noteren we na afloop.

Ten slotte vind ik het belangrijk om te noemen dat we ons realiseren dat jullie hier allemaal zitten met een eigen verhaal. Jullie hebben allemaal veel meegemaakt en ik kan me voorstellen dat hier heel veel over te zeggen valt. Omwille van de tijd kunnen we hier wellicht helaas niet altijd zo lang bij stil staan als we zouden willen. Dit komt omdat we een aantal specifieke vragen hebben die we moeten stellen. Ik zou jullie respectvol willen vragen om hier rekening mee te houden als we jullie verhaal soms wat eerder moeten afbreken.

- Zijn er nog vragen voor we beginnen?
- Dan start ik nu de video-opname

**Voorstelronde 10 minuten**

**Delen slide met voorstelvragen**

- Kun je je naam, leeftijd, en wanneer je de diagnose hebt gekregen delen?

**Kernvragen 60 minuten**

**Delen slide met thema 1.**

Dan wil ik nu starten met het eerste thema van dit gesprek, namelijk: verbetermogelijkheden in de darmkankerzorg. Bij dit onderdeel staan we ongeveer 15 minuten stil.

**Delen slide met darmkankerzorgpad**

**Topic 1. Verbetermogelijkheden darmkankerzorgpad (15 minuten)**

Als eerste ben ik benieuwd naar de verbetermogelijkheden die jullie zien in de darmkankerzorg op basis van jullie ervaringen. We denken hierover na aan de hand van het zorgpad dat de meeste mensen met darmkanker doorlopen. Hoe ziet dit pad eruit? Ter inspiratie zien jullie hier op de slide een plaatje van de verschillende fases in het zorgpad.

Van te voren hebben we jullie gevraagd om 2 of 3 ervaringen van de ontvangen zorg op te schrijven waarvan jullie dachten “dit kan toch beter”. Dit kon over elke fase in het zorgpad gaan. We geven een korte samenvatting van jullie ideeën. Uiteraard is deze samenvatting niet volledig. Na afloop van deze samenvatting zijn we benieuwd naar jullie reflecties.

*Gesprek over verbetermogelijkheden o.b.v. ervaringen*

Kunnen jullie een korte reactie geven op deze samenvatting?

**Topic 2. De rol van digitale zorg in het verbeteren van de darmkankerzorg (35 min)**

**Delen slide met thema 2**

Bedankt voor het delen van jullie ervaringen. We hebben nu een goed eerste beeld van welke verbeteringen jullie zien in het darmkankerzorgpad. Dan zou ik nu graag doorgaan met het 2^e^ thema van dit gesprek; de rol van digitale zorg in het verbeteren van de darmkankerzorg. Als eerste een vraag aan jullie:

**Delen slide met vraag**

- Waar denk je aan bij de woorden ‘digitale zorg’?

Interessant om jullie ideeën bij het begrip digitale zorg te zien. Wij houden zelf de volgende definitie van digitale zorg aan: alle informatie en communicatie technologieën die de zorg ondersteunen of verbeteren.

**Delen slide met slide voorbeelden van digitale zorg**

Om jullie eerst een beeld te geven van waar je verder allemaal aan kan denken als we het hebben over digitale zorg zie je op deze slide een aantal voorbeelden van categorieën van digitale zorg.

Nu zou ik graag met jullie in gesprek gaan over hoe digitale zorg de zorg zou kunnen verbeteren, met het oog op jullie ervaringen. Oftewel: als je terugkijkt op de zorg die je hebt ontvangen; hoe had digitale zorg dan kunnen helpen om jou een betere ervaring te geven?

Belangrijk om te noemen is dat we digitale zorg niet zien als een doel op zich, maar als een middel om de zorg te verbeteren; bijvoorbeeld om de kwaliteit te verbeteren, meer continuïteit te bieden of tijd te besparen. Wij zijn nieuwsgierig welke mogelijkheden jullie hiervoor zien.

Om jullie te helpen hierover na te denken zou ik ter inspiratie nu graag twee korte verhalen met jullie delen. In deze verhalen komen verschillende digitale zorg technologieën terug in verschillende onderdelen van het zorgpad.

**Delen slide met vragen voor bij vignettes**

Per verhaal zou ik jullie willen vragen om over twee dingen na te denken:

1) Wat vinden jullie van de manier waarop de zorg wordt aangeboden in dit verhaal?

2) Geeft dit verhaal, of een onderdeel van het verhaal, je ideeën over hoe de zaken waar jullie tegenaan liepen in jullie zorgproces, opgelost of verbeterd kunnen worden?

**Delen slide met afbeelding vignette 1 (daarna idem 2)**

***Lezen vignette 1***

**Delen slide met technologieën in zorgpadfase vignette 1**

Op deze slide zie je heel beknopt welke technologieën terugkwamen in dit verhaal en in welke fase. Dan zou ik nu graag terugkomen bij de vragen die ik jullie van te voren heb voorgelegd.

1) Wat vinden jullie van de manier waarop de zorg wordt aangeboden in dit verhaal?

2) Geeft dit verhaal, of een onderdeel van het verhaal, je ideeën over hoe de zaken waar jullie tegenaan liepen in jullie zorgpad, opgelost of verbeterd kunnen worden? Zouden deze toepassingen in een andere fase van toegevoegde waarde zijn?

*Probing vraag:
-*Zouden deze toepassingen in een andere fase van toegevoegde waarde kunnen zijn?

**Delen slide met afbeelding vignette 2**

***Lezen vignette 2*
Delen slide met technologieën in zorgpadfase vignette 2**

Op deze slide zie je heel beknopt welke technologieën terugkwamen in dit verhaal en in welke fase. Dan zou ik nu graag terugkomen bij de vragen die ik jullie van te voren heb voorgelegd.

1) Wat vinden jullie van de manier waarop de zorg wordt aangeboden in dit verhaal?

2) Geeft dit verhaal, of een onderdeel van het verhaal, je ideeën over hoe de zaken waar jullie tegenaan liepen in jullie zorgpad, opgelost of verbeterd kunnen worden?

*Probing vraag:
-*Zouden deze toepassingen in een andere fase van toegevoegde waarde kunnen zijn?

Na de vignettes:

**Delen slide met voorbeelden digitale zorg**

We hebben nu jullie ideeën besproken aan de hand van de vignettes. Ten slotte wil ik jullie nogmaals de slide met voorbeelden van digitale zorg laten zien.

3) Denk nog eens terug aan jullie eigen ervaringen. Hebben jullie nog andere ideeën hoe één van deze technologieën de zorg kunnen verbeteren?

*Probing vraag:*- Als ik kijk naar de slide met jullie eigen ervaringen werden daar ook verbetermogelijkheden genoemd die (u*itkiezen waar nog weinig over gesproken is):
**binnen de nazorgfase vallen /
*te maken hebben met communicatie en de relatie met de arts genoemd/
*over ‘het omgaan met de ziekte’ en ‘revalidatie’ gaan;
Zou digitale zorg daar nog een rol kunnen spelen?

**Afsluiting 5 minuten**

*We hebben nu heel mooi een overzicht gekregen van verbetermogelijkheden binnen de darmkankerzorg en de kansen voor digitale zorg.*

- Zijn er nog punten die we niet besproken hebben, die jullie kwijt willen?
- Wat vonden jullie van het gesprek?

**Vervolg en dank 5 minuten**

*We willen jullie nogmaals hartelijk danken voor jullie medewerking.*

*We zullen de gesprekken anoniem gaan verwerken en analyseren. De resultaten worden gepubliceerd in een wetenschappelijk artikel. Zijn er onder jullie mensen die interesse hebben om dit artikel toegestuurd te krijgen?*

*De cadeaubon zullen we je binnenkort toesturen via de mail.

Als je nog vragen of inhoudelijke toevoegingen achteraf hebt, mag je altijd contact met ons opnemen. Mijn mailadres staat op de slide.*
