# Supplementary material for: Exploring colorectal cancer survivors’ perspectives on improving care delivery and the role of e-health technology: a qualitative study
Source: Support Care Cancer. 2023 Aug 31;31(9):544. doi: 10.1007/s00520-023-08007-8 (PMC10471668; doi:10.1007/s00520-023-08007-8)
Supplement: Supplementary file 2 — CRC care pathway phases (DOCX 28 KB) [file 520_2023_8007_MOESM2_ESM.docx]

**Table 1.** Overview of the different phases of the CRC care pathway (original source: van Deursen et al., 2023^1^)

| **Phase** | **Description** |
| --- | --- |
| Referral | Patients are referred to a hospital based on national population screening test results or if a general practitioner suspects CRC. |
| Diagnosis | The patient undergoes one or more tests to locate the tumor and determine the type and growth rate and whether there are metastases. An intake interview and an endoscopy or an examination of the large intestine using an endoscope are usually conducted. |
| Treatment | Based on the diagnosis, treatment options are discussed with the patient. Examples of treatment options are surgery or chemotherapy. Patients often follow a pre-habilitation program to prepare for surgery (e.g., physical training, advice on nutrition and mental support). |
| Aftercare | When (part of) the treatment is completed, patients are supported in their recovery, and any complications are monitored. Referral (i.e., to social workers or physiotherapists) can occur based on monitoring physical and psychological health. |
| Palliative care | Patients who cannot recover from CRC receive care to optimize quality of life. It consists of, among other things, pain relief and (psycho) social support. Advance Care Planning (ACP) is used to discuss the wishes and needs of patients with a healthcare provider. |

**References**

1. van Deursen L, van der Vaart R, Alblas EE, et al: Improving the colorectal cancer care pathway via e-health: a qualitative study among Dutch healthcare providers and managers. Supportive Care in Cancer 31:203, 2023
