# Supplementary material for: Exploring colorectal cancer survivors’ perspectives on improving care delivery and the role of e-health technology: a qualitative study
Source: Support Care Cancer. 2023 Aug 31;31(9):544. doi: 10.1007/s00520-023-08007-8 (PMC10471668; doi:10.1007/s00520-023-08007-8)
Supplement: Supplementary file 3 — Description of e-health categories (DOCX 25 KB) [file 520_2023_8007_MOESM3_ESM.docx]

Supplementary file 3. Description of e-health categories

**Digital communication** – the use of technology to remotely communicate with your healthcare provider. This can be done through video calling, secured e-mail or chat, among other methods.

**Online information resources** – websites and apps that allow you to access information about your health or treatment on your own.

**Telemonitoring** – technology that allows you to measure your health values, such as temperature, heart rate, and blood pressure, or complete digital questionnaires from the comfort of your own home as part of your treatment policy. This way, your healthcare provider can remotely monitor your health.

**Self-monitoring** – apps and wearables to measure your health values and gain insights into your well-being. These tools can also provide lifestyle advice. The data is not automatically shared with your healthcare provider.

**Patient portal** – a secured website or application that allows you to access and view your personal medical records. You can also use it to schedule appointments and manage other practical aspects of your healthcare.
