# Supplementary material for: Exploring colorectal cancer survivors’ perspectives on improving care delivery and the role of e-health technology: a qualitative study
Source: Support Care Cancer. 2023 Aug 31;31(9):544. doi: 10.1007/s00520-023-08007-8 (PMC10471668; doi:10.1007/s00520-023-08007-8)
Supplement: Supplementary file 4 — Vignettes (DOCX 493 KB) [file 520_2023_8007_MOESM4_ESM.docx]

Supplementary file 4. Vignettes

**Vignette 1**


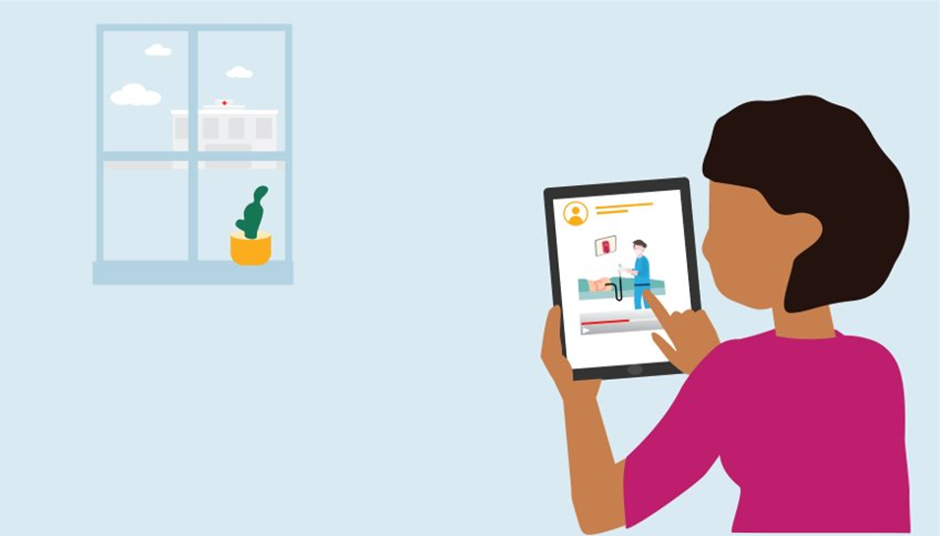


*English translation*
Karin is a 58-year-old woman. She recently went to her doctor because she had blood in her stools and had lost a lot of weight in a short amount of time. Her doctor referred her to the hospital. Here, she will soon have an appointment to view the intestines (an endoscopy). The hospital doctor has asked her whether she wants to do the intake for the endoscopy digitally or whether she wants to come to the outpatient department. Karin has opted for the digital intake. This means that, she receives information about the endoscopy via videos, for example, about the preparations required (such as not eating or drinking prior to the endoscopy) and the risks involved. These videos can be watched on her personal page in the hospital's patient portal. Here, she can also fill out a digital questionnaire. This questionnaire includes questions about her medication use and medical history, such as her previous illnesses. Based on this questionnaire, the doctor determines if she must come for an intake consultation at the hospital or if she can come immediately for the endoscopy. Since Karin does not feel digitally skilled, the nurse explained that she could contact a digital counter by telephone if she had any questions.

*Original text [in Dutch]*
Karin is een vrouw van 58 jaar. Onlangs is zij naar haar huisarts gegaan omdat ze bloed bij haar ontlasting had en in korte tijd veel was afgevallen. Haar huisarts heeft haar doorverwezen naar het ziekenhuis. In het ziekenhuis heeft ze binnenkort een afspraak voor een kijkonderzoek in de darm (een colonscopie). De arts van het ziekenhuis heeft haar de keuze gegeven of ze de intake voor de scopie digitaal wil doen of dat ze naar de poli wil komen. Karin heeft gekozen voor de digitale intake. Dit houdt in dat zij via filmpjes uitleg krijgt over het kijkonderzoek, bijvoorbeeld over de voorbereiding die nodig is (zoals niet eten en drinken voorafgaand aan het kijkonderzoek) en de risico’s. Deze filmpjes kan ze vinden op haar persoonlijke pagina in het patiëntportaal van het ziekenhuis. Hier kan zij ook een digitale vragenlijst invullen. In deze vragenlijst staan vragen over onder andere haar medicatiegebruik en medische voorgeschiedenis, zoals eerdere ziektes die ze heeft gehad. Op basis van deze vragenlijst bepaalt de arts of het nodig is dat zij nog op consult moet komen in het ziekenhuis of dat ze direct langs kan komen voor het kijkonderzoek . Aangezien Karin zich niet digitaal vaardig voelt heeft de verpleegkundige haar uitgelegd dat ze telefonisch contact kan opnemen met een digitaal loket als ze vragen heeft.

**Vignette 2**


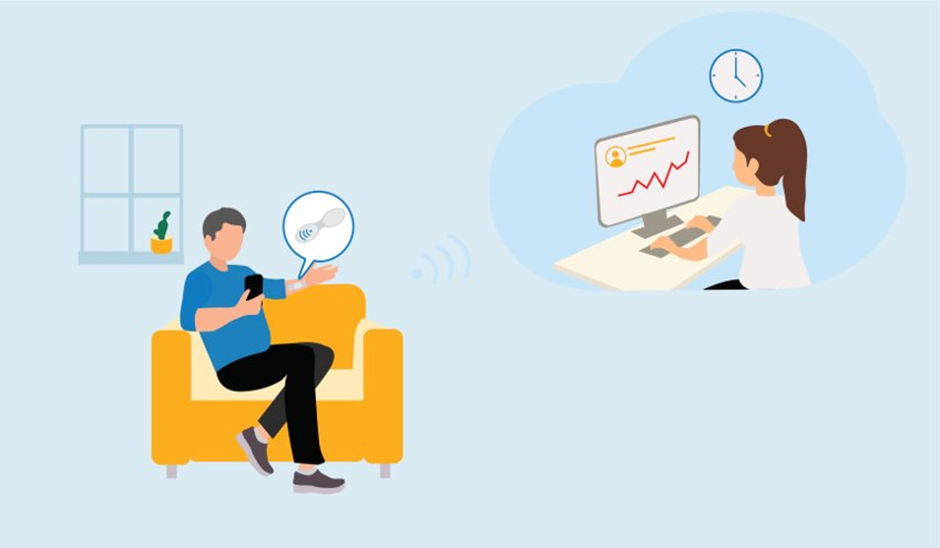


*English translation*
Bert de Vries is a 61-year-old man. He was diagnosed with colon cancer and had surgery last week. He was allowed to leave the hospital earlier because the doctor could monitor him at home via telemonitoring. Bert wears a so-called smart patch for this. This patch automatically transmits his heartbeat and temperature to the hospital. This way, Bert's health is continuously monitored without him noticing this. This lasts for ten days. During these ten days, Bert also daily completes a short digital questionnaire on his smartphone via an app. The questions are about pain, eating and drinking, exercising, and how he feels. His answers are automatically sent to the doctor. If there are concerns, the doctor will contact Bert. The app also automatically advises Bert based on his answers, for example, to take a short walk more often or to eat more fiber-rich food.

*Original text [in Dutch]*
Bert de Vries is een man van 61 jaar. Hij heeft darmkanker en is vorige week geopereerd. Hij mocht eerder naar huis uit het ziekenhuis, omdat de arts thuis met hem mee kan kijken hoe het gaat, via telemonitoring. Hiervoor draagt Bert een zogenaamde slimme pleister. Deze pleister geeft automatisch de hartslag en temperatuur van Bert door aan het ziekenhuis. Op deze manier wordt continu in de gaten gehouden of alles goed met hem gaat, zonder dat Bert dit merkt. Dit duurt 10 dagen. Tijdens deze 10 dagen vult Bert ook elke dag een korte digitale vragenlijst op zijn mobiel in, via een app. De vragen gaan over zaken zoals pijn, eten en drinken, bewegen en hoe hij zich voelt. Zijn antwoorden worden automatisch naar de arts verstuurt. Mocht er iets niet in orde zijn, dan neemt de arts contact op met Bert. Ook geeft de app automatisch advies aan Bert op basis van zijn antwoorden, bijvoorbeeld om wat vaker een korte wandeling te maken, of wat vaker vezelrijk voedsel te eten.
